# Comparison of Gut Microbiota Diversity Between Captive and Wild Tokay Gecko (*Gekko gecko*)

**DOI:** 10.3389/fmicb.2022.897923

**Published:** 2022-06-17

**Authors:** Sanqi Tang, Yuhui Li, Chengming Huang, Shufa Yan, Yongtai Li, Zening Chen, Zhengjun Wu

**Affiliations:** ^1^Key Laboratory of Ecology of Rare and Endangered Species and Environmental Protection (Guangxi Normal University), Ministry of Education, Guilin, China; ^2^Guangxi Key Laboratory of Rare and Endangered Animal Ecology, Guangxi Normal University, Guilin, China; ^3^Key Laboratory of Animal Ecology and Conservation, Institute of Zoology, Chinese Academy of Sciences, Beijing, China

**Keywords:** *Gekko gecko*, captive population, 16S rRNA, gut microbiota, wildlife, conservation, microbial community diversity

## Abstract

Captive animals and wild animals may exhibit different characteristics due to the heterogeneity of their living environments. The gut microbiota play an important role in the digestion and absorption, energy metabolism, immune regulation, and physiological health of the host. However, information about the gut microbiota of captive and wild *Gekko gecko* is currently limited. To determine the difference in gut microbiota community composition, diversity, and structure between captive and wild geckos, we used the Illumina miseq platform to conduct high-throughput sequencing and bioinformatics analysis of the v3–v4 hypervariable region of 16S rRNA in 54 gecko samples. Our results showed that Proteobacteria, Firmicutes, Bacteroidetes, and Actinobacteria were the dominant gut microbiota phyla of the gecko. The dominant genera comprised mainly *Pseudomonas*, *Burkholderia-caballeronia-paraburkholderia*, *Ralstonia*, *Romboutsia*, and *Bacteroides*. Captive geckos had significantly higher alpha diversity and potential pathogenic bacteria than wild populations. Moreover, significant differences in beta diversity of gut microbiota were observed between two populations. Functional prediction analysis showed that the relative abundance of functional pathways of wild geckos was more higher in metabolism, genetic information processing and organismal system function than those in captive geckos. Total length significantly affected gut microbial community (R^2^ = 0.4527, *p* = 0.001) and explained 10.45% of the total variation for gut microbial community variance between two groups. These results may be related to differences in diet and living environment between two populations, suggesting that the management of captive populations should mimic wild environments to the greatest extent possible to reduce the impact on their gut microbiota.

## Introduction

The gut microbiota has symbiosis and coordinated evolution with the host, forming an overall system of interaction (Nicholson et al., 2012; Tremaroli and Bäckhed, 2012; Flint et al., 2015). The gut microbiota plays important roles in host growth and development (Egert et al., 2006; Sommer and Bäckhed, 2013), impacting energy budget (Semova et al., 2012), nutritional metabolism (Cani and Everard, 2016; Greer et al., 2016), vitamin synthesis (LeBlanc et al., 2013), digestion and absorption, colonization resistance and immune homeostasis (Hooper et al., 2012; Bengmark, 2013), and behavior and emotion (Heijtz et al., 2011; Ezenwa et al., 2012). At the same time, various factors also affect the gut microbiota, including host diet (Ley et al., 2008; De Filippo et al., 2010; Muegge et al., 2011; Wu et al., 2011), genetic and environmental history (Moeller et al., 2013), physiological status (Amato et al., 2013; McCord et al., 2014), and external environmental factors. Studies on human gut microbiota have identified the relationship between gut microbiota disorders and host infections with opportunistic pathogens (Shin et al., 2015), allergies (Bisgaard et al., 2011), and diseases (Ley et al., 2006; Sobhani et al., 2011; Vaarala, 2011).

Currently, the gut microbiota has been widely studied in invertebrates (Clark and Walker, 2018), amphibians (Bletz et al., 2016) and reptiles (Trevelline et al., 2019; [Bibr B74][Bibr B19]), mammals (Hu et al., 2017; Song et al., 2017; Li et al., 2021), and birds (Ryu et al., 2012; Waite et al., 2012; Grond et al., 2018). In recent years, research into the gut microbiota in captive animals, such as *Takydromus septentrionalis* (Zhou et al., 2020), *Neotoma albigula* (Martínez-Mota et al., 2020), and *Macaca mulatta* (Chen et al., 2019) has also attracted extensive attention. The gut microbiota of captive animals may differ from those of wild animals due to dietary differences, antibiotic treatment, human activities, and exposure to other species in captivity (Alfano et al., 2015; Clayton et al., 2016; Chen et al., 2019; Eliades et al., 2021). Studies on mammals and reptiles—such as *Hydrorga leptonyx* (Nelson et al., 2013), *Peromyscus maniculatus* (Schmidt et al., 2019), *Macaca mulatta* (Chen et al., 2019) and *Shinisaurus crocodilurus* (Jiang et al., 2017), *Crocodylus siamensis* (Lin et al., 2019), and *Brachylophus vitiensis* (Eliades et al., 2021)—have confirmed that captivity may change the composition and abundance of the gut microbiota. Moreover, captivity may lead to an increase in potential pathogens in the animal gut microbiota, leading to an increased incidence of disease (Berry et al., 2012; Amato et al., 2016; Kohl et al., 2016; Xie et al., 2016). Conversely, a few studies reported that captivity may improve host immune status (Montalban-Arques et al., 2015), and even have beneficial effects on the development and behavior of the host (Heijtz et al., 2011). Therefore, an evaluation of the effects of captivity on the gut microbiota of wild animals in conservation and rescue breeding is necessary (Eliades et al., 2021).

In China, Tokay geckos are distributed mainly in Guangdong province, Guangxi Zhuang autonomous region, Yunnan province, Fujian province, and Taiwan (Huang et al., 1995; Tang et al., 1997). Geckos are used in traditional Chinese medicine (Li et al., 1996; Bauer, 2009). While medical demand for geckos is increasing, the captive breeding population cannot meet the market demand (Li et al., 1996). In addition, the natural habitat of geckos has been destroyed and gradually reduced, affecting their survival (Zhang et al., 2015). These factors have caused a decline in wild gecko populations, rendering *ex-situ* conservation the effective conservation approach for this species. Due to the heterogeneity of captive and natural environments, however, captive and wild geckos may differ greatly in diet composition. Moreover, captive populations are exposed to a greater extent to human activity than wild populations. The effect of different living environments on the gut microbiota of geckos remains unclear. We therefore analyzed the gut microbiota community of captive and wild geckos by 16S rRNA gene sequencing technology, and compared the microbial community diversity, richness, structure, and function of the two groups. Considering the differences in living environment between wild animals and captive animals, we propose the following predictions: (1) wild geckos have a higher diversity of gut microbiota than captive geckos and (2) the gut microbiota of captive geckos include more opportunistic pathogens.

## Materials and Methods

### Sample Collection

We collected a total of 54 samples, including 24 from wild geckos (18 males and 6 females) and 30 from captive geckos (22 males and 8 females). We captured the wild geckos in Jiangzhou District, Chongzuo City, Guangxi, and acquired the captive geckos from Nanning Junhao Wildlife Science and Technology Development Co., Ltd. Wild geckos eat mainly insects and moths from their natural environment (Liu et al., 1981; Tang et al., 1997). Captive individuals are fed on *Zophobas morio* and *Tenebrio molitor* and kept in temperature conditions of about 20°C. Average total length (TL) of wild and captive geckos is 233.92 ± 38.93 mm and 266.38 ± 32.82 mm, respectively. All Tokay geckos were healthy during the sampling period (Supplementary Table 1).

We used cloacal swabs—an acceptable source for non-destructive sampling of the gut microbiota of reptiles (Colston et al., 2015; Jiang et al., 2017)—to collect the gut microbiota of geckos. After sampling, we released all individuals back into the site of capture. We stored samples in a thermal insulation bucket with ice bags and transported them to a refrigerator at –20°C for storage.

### DNA Extraction, PCR Amplification, and Sequencing

We extracted the total gut microbiota community genomic DNA from all samples using the FastDNA^®^ Spin Kit for Soil (MP Biomedicals, United States) based on the manufacturer’s instructions. We detected the extraction quality of DNA by 1% agarose gel electrophoresis and determined the concentration and purity of DNA with a NanoDrop 2000 UV-vis spectrophotometer (Thermo Fisher Scientific, Wilmington, DE, United States). We amplified the hypervariable region v3–v4 of the bacterial 16S rRNA gene with primer pairs 338F (5′-ACTCCTACGGGAGGCAGCAG-3′) and 806R (5′-GGACTACHVGGGTWTCTAAT-3′) by an ABI GeneAmp^®^ 9700 polymerase chain reaction (PCR) thermocycler (ABI, CA, United States) (Mori et al., 2014; Chen et al., 2019). Our PCR reaction parameters included initial denaturation at 95°C for 3 min, followed by 27 cycles of denaturation at 95°C for 30 s, annealing at 55°C for 30 s and extension at 72°C for 45 s, and ended with a final extension at 72°C for 10 min. For the PCR test we used TransGen AP221-02: TransStart Fastpfu DNA Polymerase with 20 μL of reaction system containing 5 × FastPfu Buffer 4 μL, 2.5 mM deoxy-ribonucleoside triphosphates (dNTPs) 2 μL, Forward Primer (5 μM) 0.8 μL, Reverse Primer (5 μM) 0.8 μL, FastPfu Polymerase 0.4 μL, bovine serum albumin (BSA) 0.2 μL, template DNA 10 ng, and finally ddH_2_O up to 20 μL. We performed PCR reactions in triplicate. We extracted the PCR product from 2% agarose gel, purified it using the AxyPrep DNA Gel Extraction Kit (Axygen Biosciences, Union City, CA, United States) according to manufacturer’s instructions, and quantified it using Quantus™ Fluorometer (Promega, United States). We pooled purified amplicons in equimolar and paired-end sequenced them (2 × 300) on an Illumina miseq platform (Illumina, San Diego, CA, United States) according to the standard protocols by Majorbio Bio-Pharm Technology Co. Ltd. (Shanghai, China).

### Processing of Sequencing Data and Quality Evaluation

We demultiplexed the raw 16S rRNA gene sequencing reads, quality-filtered them by Trimmomatic, and merged them by FLASH with the following criteria: (i) we truncated the 300 bp reads at any site receiving an average quality score of <20 over a 50 bp sliding window, and discarded both the truncated reads shorter than 50 bp and reads containing ambiguous characters; (ii) we assembled only overlapping sequences longer than 10 bp according to their overlapped sequence; we adopted a maximum mismatch ratio of overlap region of 0.2; we discarded reads that could not be assembled; (iii) we distinguished samples according to the barcode and primers, and adjusted the sequence direction, exact barcode matching, and 2 nucleotide mismatches in primer matching.

We obtained a total of 2,603,017 raw sequences from the v3–v4 region of the hypervariable region of 16S rRNA gene in 54 samples. After quality control, 2,504,574 sequences were effective (average ± SD = 48,204.02 ± 9,796.15 for each sample), and the average length of the sequences was 420.66 ± 7.13 bp (Supplementary Table 2). Our rarefaction curve tended to be flat, indicating that the amount of sequencing was reasonable, and the sequencing depth was sufficient (Supplementary Figure 1). The Good’s coverage estimates of the 54 samples ranged from 98.89% to 99.91%, indicating that we had identified almost all bacterial communities in the samples (Table 1).

**TABLE 1:** Alpha diversity index of the gut microbiota in Tokay gecko.

Estimators	Captive	Wild	*p* value
Shannon	2.686 ± 0.603	1.930 ± 1.240	0.004
Simpson	0.207 ± 0.088	0.398 ± 0.278	0.025
Ace	494.530 ± 111.510	401.820 ± 304.350	0.001
Chao	497.870 ± 109.330	383.370 ± 301.540	<0.001
Good’s coverage	0.998 ± 0.001	0.997 ± 0.002	0.577

### Bioinformatics Analysis

We clustered operational taxonomic units (OTUs) with 97% similarity cutoff (Edgar, 2013) using UPARSE (version 7.0.1090^1^) and identified and removed chimeric sequences using UCHIME. We analyzed the taxonomy of each OTU representative sequence by RDP Classifier (version 2.11^2^) against the 16S rRNA database (for instance, Silva Release138^3^) using a confidence threshold of 70%. We used rarefaction curves to reflect whether sequencing data was reasonable, and the coverage index to indicate the real situation of sequencing results. We used the Mothur program to calculate the alpha diversity index to reflect community diversity (Shannon index and Simpson index) and community richness (Abundance-based Coverage Estimator (Ace) and Chao index) (version v.1.30.2^4^). We used the Bray–Curtis distance algorithm for sample hierarchical cluster analysis of gut microbiota of captive and wild geckos and assessed beta diversity by principal coordinate analysis (PCoA) based on weighted and unweighted UniFrac distance metrics using QIIME (version 1.9.1^5^). Adonis permutational multivariate analysis (Adonis/PERMANOVA) was performed to evaluate the dissimilarity among samples with permutation set at 999. We used the Wilcoxon rank-sum test to analyze gut microbiota diversity differences between wild and captive groups and adjusted the *p* values under the control of FDR level at 0.05. We performed linear discriminant analysis (LDA) effect size (LEfSe) analyses to identify potential microbial biomarkers between groups.^6^ For the LDA interpretation, we considered differences as significant for a *p* < 0.05 and an LDA score >4. We predicted functional profiles of microbial communities using PICRUSt2 (phylogenetic investigation of communities by reconstruction of unobserved states 2; Langille et al., 2013) (version 2.2.0^7^). We performed the Wilcoxon rank-sum test to test the differences of functional pathways between the two groups in the Kyoto Encyclopedia of Genes and Genomes (KEGG) using IBM SPSS (version 23.0), and the threshold on the *p* value was set at 0.05. Detrended correspondence analysis (DCA) was conducted to obtain the length of first axis (R version 3.3.1, vegan package), RDA (Redundancy analysis), or CCA (Canonical correlation analysis) was chosen based on the value of DCA 1 (>4, CCA; <3 RDA; 3–4 RDA/CCA). The DCA analysis showed that the length of DCA 1 was 6.05, indicating a unimodal-model-based CCA analysis was more suitable than RDA analysis to test the effect of geckos’ total length and sex on microbial community at OTU level. A variation partitioning analysis (VPA) was conducted to examine the contribution of total length and sex factors in influencing microbial community structure as determined by CCA analysis (R version 3.3.1, vegan package). Further, the partial Mantel test was used to detect the Bray–Curtis distance matrix correlation between total length and sex and microbial community with 999 permutations [QIIME version 1.9.1 (see footnote 5)]. We used the non-parametric Spearman correlation test to analyze the correlation between total length and sex of geckos and the relative abundance of the top 50 microbial genera (R version 3.3.1, pheatmap package).

## Results

### Differences in Alpha and Beta Diversity of Gut Microbiota

**FIGURE 1:**
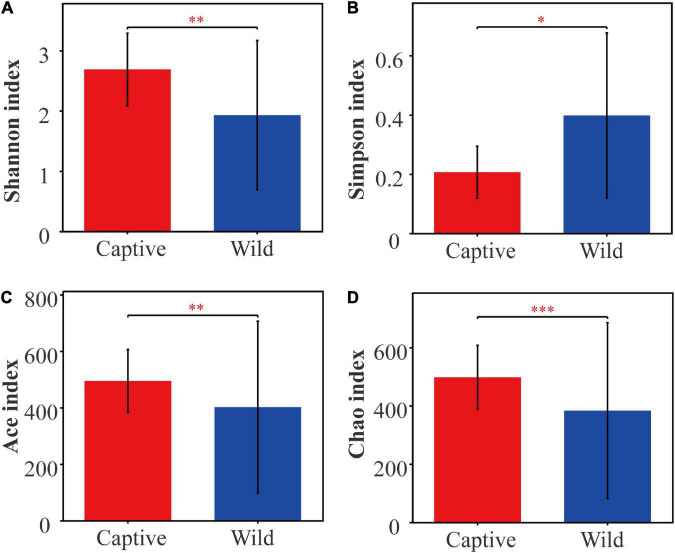
Alpha diversity of gut microbiota between captive and wild geckos. **(A)** Shannon index; **(B)** Simpson index; **(C)** Ace index; **(D)** Chao index. Significant difference 0.01 < *p* ≤ 0.05 was marked as “*”, 0.001 < *p* ≤ 0.01 was marked as “**”, and *p* ≤ 0.001 was marked as “***”.

Alpha diversity analysis showed that there were significant differences in community richness and diversity (*p* < 0.05; Table 1); captive geckos had higher Ace, Chao, and Shannon indices and a lower Simpson index than those of wild geckos (Table 1 and Figure 1).

**FIGURE 2:**
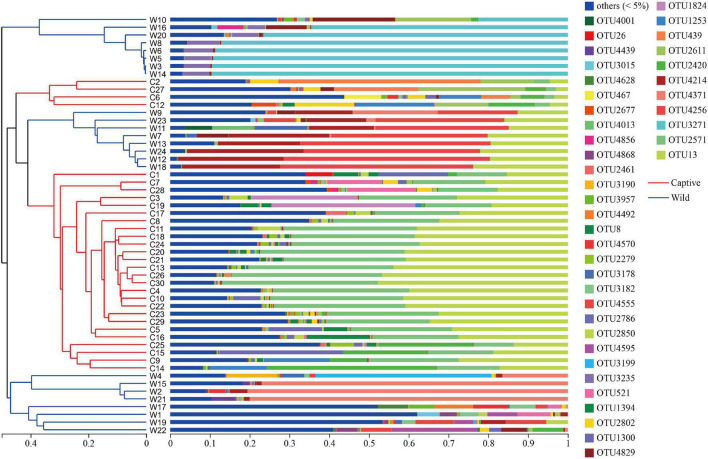
Hierarchical cluster analysis based on Bray–Curtis distance algorithm at the OTU level.

**FIGURE 3:**
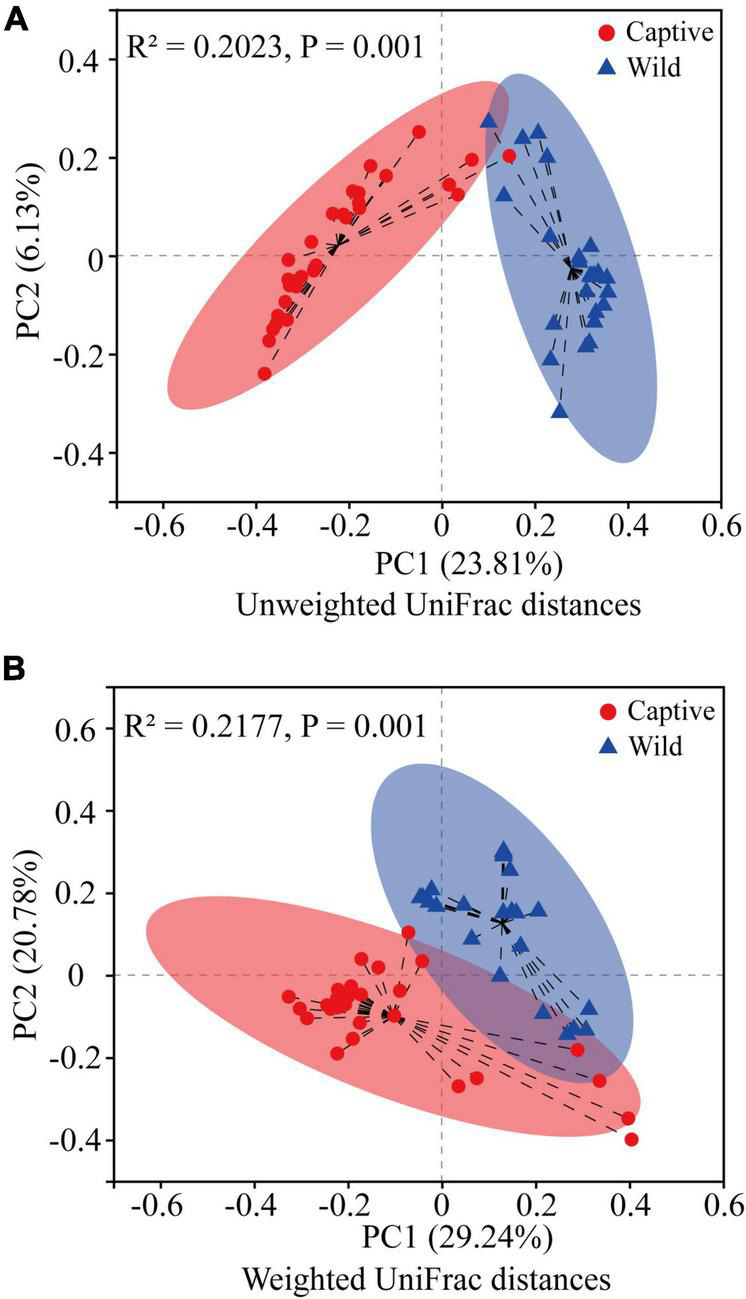
Difference in gut microbiota community structure between captive and wild geckos using Adonis test (permutation = 999). **(A)** A principal coordinate analysis of unweighted UniFrac distances; **(B)** A principal coordinate analysis of weighted UniFrac distances.

Hierarchical cluster analysis based on Bray–Curtis distance algorithm showed that the wild and captive geckos divided into five small branches in total, without making two specific clusters according to groups (Figure 2). Our analysis clustered captive geckos in two branches, and wild geckos in three smaller branches. PCoA based on unweighted and weighted UniFrac distances revealed high clustering of the gut microbiota according to group, highlighting a significant separation between captive and wild geckos (Unweighted UniFrac: R^2^ = 0.2023, *p* = 0.001; Weighted UniFrac: R^2^ = 0.2177, *p* = 0.001; Figure 3).

### Differences in the Composition and Abundance of Gut Microbiota

**FIGURE 4:**
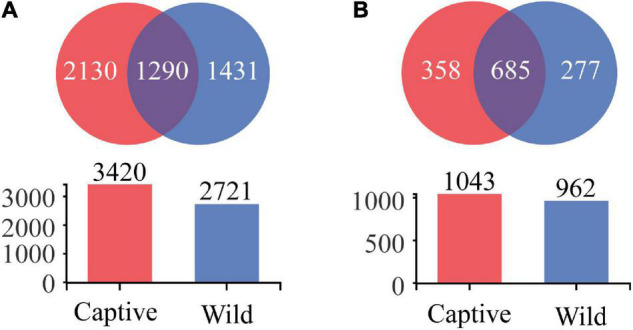
Venn diagram of gut microbiota in Tokay gecko at the OTU **(A)** and genus **(B)** level.

We obtained 4,851 OTUs at 97% sequence similarity and classified them into 52 phyla, 153 classes, 353 orders, 586 families, and 1,320 genera. Of the 4,851 OTUs, 1,290 OTUs were shared by captive and wild geckos, whereas 2,130 OTUs and 1,431 OTUs were unique to captive geckos and wild geckos, respectively (Figure 4A). At the genus level, a total of 685 genera were shared by captive and wild geckos, with 358 genera specific to the captive group and 277 genera specific to the wild group (Figure 4B).

**FIGURE 5:**
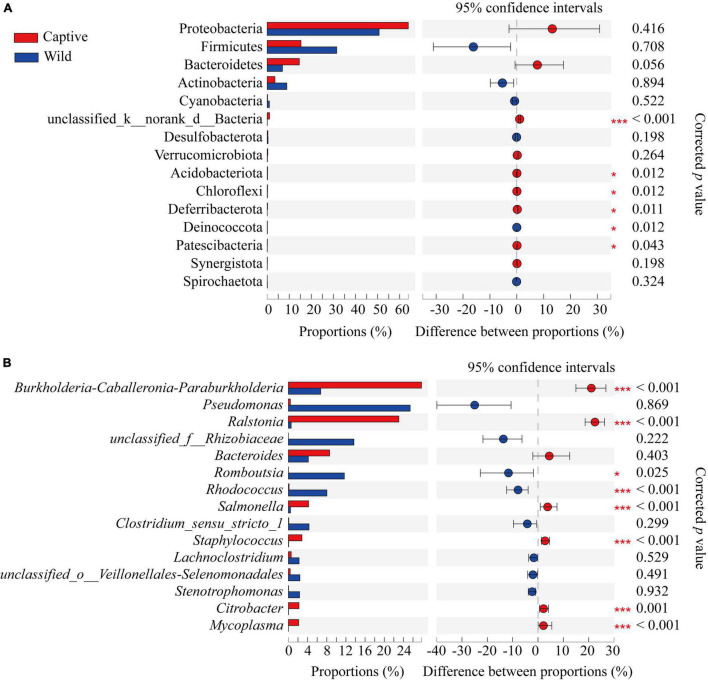
Differences in gut microbiota composition between captive and wild geckos at the phylum level **(A)** and genus level **(B)** of the top 15 taxa. Significant difference 0.01 < *p* ≤ 0.05 was marked as “*”, and *p* ≤ 0.001 was marked as “****”.

At the phylum level, Proteobacteria were the predominant phylum in two groups (captive: 63.88% ± 22.99%; wild: 50.67% ± 36.90%), followed by Firmicutes (captive: 15.29% ± 11.18%; wild: 31.45% ± 33.94%), Bacteroidetes (captive: 14.55% ± 22.11%; wild: 6.90% ± 9.90%), and Actinobacteria (captive: 3.51% ± 2.96%; wild: 8.87% ± 10.41%), respectively; Wilcoxon rank-sum test showed that the relative abundance of Acidobacteriota, Chloroflexi, Deferribacterota, and Patescibacteria was higher in the captive geckos than those in wild geckos (Figure 5A; Supplementary Table 3).

At the genus level, the main gut microbiota included *Burkholderia-caballeronia-paraburkholderia* (captive: 27.77% ± 13.56%; wild: 6.73% ± 8.94%), *Ralstonia* (captive: 23.05% ± 11.21%; wild: 0.59% ± 0.47%), *Pseudomonas* (captive: 0.41% ± 0.76%; wild: 25.40% ± 38.72%), and *Bacteroides* (captive 8.59% ± 18.71%; wild: 4.16% ± 6.38%); Wilcoxon rank-sum test showed that the relative abundance of *Romboutsia* and *Rhodococcus* was higher in wild geckos, and the relative proportion of *Burkholderia-caballeronia-paraburkholderia*, *Ralstonia*, *Salmonella*, *Staphylococcus*, *Citrobacter*, and *Mycoplasma* was higher in captive geckos (Figure 5B and Supplementary Table 4).

**FIGURE 6:**
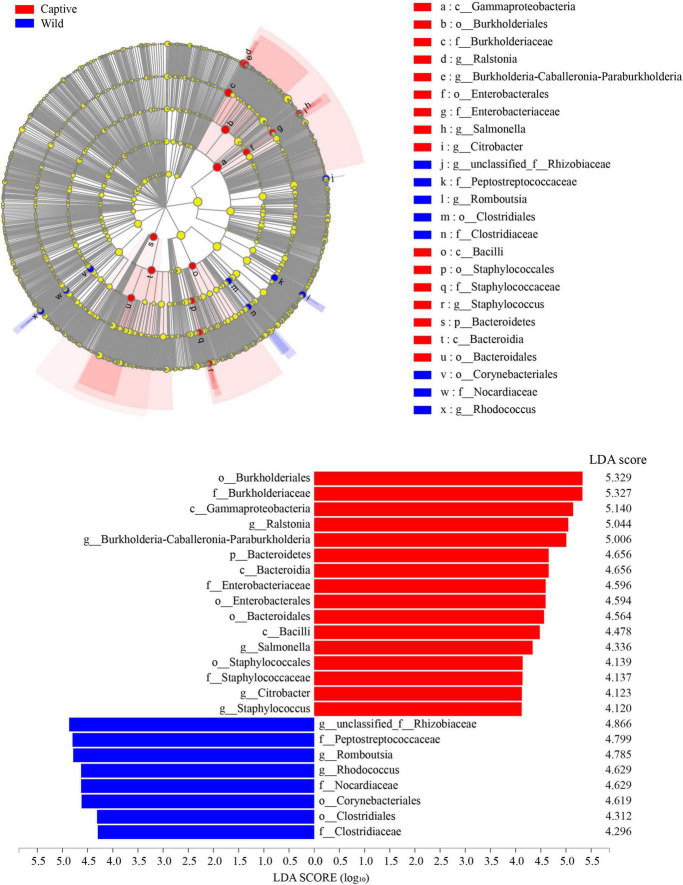
LEfSe (LDA Effect Size) analysis of the gut microbiota in Tokay gecko (LDA score > 4.0).

LEfSe analysis (LDA score > 4.0) showed that the order Burkholderiales, the family Burkholderiaceae, and the class Gammaproteobacteria were the most important taxa contributing to gut microbiota differences between captive and wild geckos (Figure 6).

### Gut Microbiota Functional Profile Prediction

**FIGURE 7:**
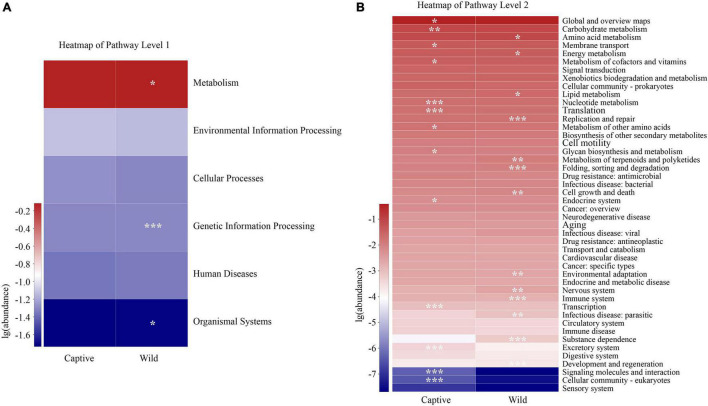
Differences in the functional profiles in level 1 **(A)** and level 2 **(B)** pathways of the gut microbiota in Tokay gecko. The *p* value was represented by asterisks. Significant difference 0.01 < *p* < 0.05 was marked as “*”, 0.001 < *p* < 0.01 was marked as “**”, and *p* < 0.001 was marked as “***”.

Our results identified significant differences between two groups in three pathways at the KEGG pathway level 1 (Wilcoxon rank-sum test, *p* < 0.05), including those in metabolism, genetic information processing, and organismal systems, with a greater relative abundance in wild geckos than in captive geckos (Figure 7A). We detected 46 functional pathways at the KEGG pathway level 2, among which we identified significant differences in 27 pathways between captive and wild samples. The relative abundance of amino acid metabolism, energy metabolism and lipid metabolism in the gut microbiota of wild samples were higher than those of the captive population. However, we detected significant enrichment in global and overview maps, carbohydrate metabolism, and membrane transport in captive samples (Figure 7B).

### Effects of Total Length and Sex on Gut Microbiota of Tokay Gecko

**FIGURE 8:**
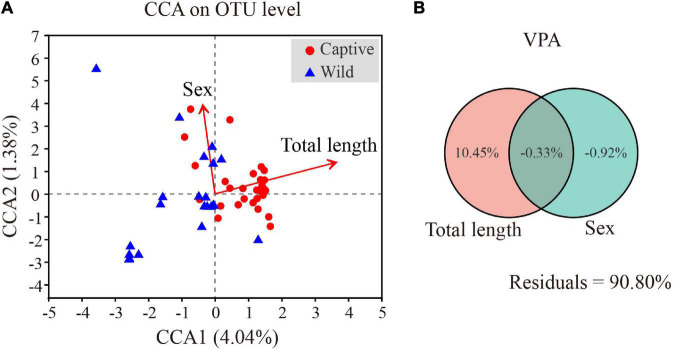
Effects of total length and sex on gut microbiota of Tokay gecko **(A)**. Canonical correlation analysis of the microbial community on OTU level and physical parameters of Tokay gecko **(B)**.

Canonical correlation analysis results showed the first two axes explained 5.42% of total variance (CCA1: 4.04%; CCA2: 1.38%). Total length (R^2^ = 0.4527, *p* = 0.001) and sex (R^2^ = 0.3705, *p* = 0.001) significantly affected gut microbial community (Figure 8A). VPA demonstrated that total length of Tokay geckos explained 10.45% of the total variation for gut microbial community variance (Figure 8B). A partial Mantel test showed that total length was weakly and positively correlated with gut microbial community (*r* = 0.147, *p* = 0.019, conditioning on sex), whereas no significant correlation was observed between sex and gut microbial community (*r* = 0.004, *p* = 0.421, conditioning on total length). Spearman correlation showed that total length was significantly positively correlated with *Burkholderia-Caballeronia-Paraburkholderia* (*r* = 0.558, *p* < 0.001), *Ralstonia* (*r* = 0.478, *p* < 0.001), and *Staphylococcus* (*r* = 0.309, *p* = 0.023) (Supplementary Table 5).

## Discussion

In terms of microbial composition, Proteobacteria, Firmicutes and Bacteroidetes were the most dominant phyla in both captive and wild geckos, suggesting these microbes may play an important role in maintaining relative stability of gut microbiome. Other studies have also shown these microbes to be the main dominant phyla among vertebrate gut microbiota, including *Moschus berezovskii* and *Moschus chrysogaster* (Hu et al., 2017), *Ciconia boyciana* (Wu et al., 2021), *Naja atra*, *Ptyas mucosus*, *Elaphe carinata*, and *Deinagkistrodon acutus* (Zhang et al., 2019). However, we observed significant differences in microbial community structure between the captive and the wild geckos (Figures 2, 3). We also discovered a higher alpha diversity of gut microbiota in captive geckos than that in wild geckos, which was inconsistent with our prediction 1. Similar results have been found in several species, including *Takifugu bimaculatus* (Lei et al., 2020), *Shinisaurus crocodilurus* (Tang et al., 2020), *Psittaciformes* (Xenoulis et al., 2010), and *Macaca mulatta* (Chen et al., 2019). Moreover, captive geckos contained a larger total number of OTUs and a greater abundance of unique OTUs. The captive environment increased human contact, individual interaction, and drug use (McKenzie et al., 2017), which might allow a greater microbial diversity and more distinct taxa to colonize the intestinal tract of captive geckos. In addition, differences in dietary composition and quantities may also play an important role (De Filippo et al., 2010; Jiang et al., 2017). Captive geckos feed mainly on locusts, woodlouse worms and barley worms, whereas wild geckos eat mainly insects and moths (Liu et al., 1981; Tang et al., 1997). Moreover, captive geckos are fed adequate food at regular intervals, but wild geckos have erratic diets because of their need to search for natural food and the fluctuations in weather or habitat conditions. Therefore, although the gut microbiota of geckos inhabiting different living environments did not differ in their most dominant phyla, the diversity and structure of the microbial community differed significantly, indicating that the captive environment had an obvious influence on gecko gut microbiota.

At the genus level, we detected a higher relative abundance of *Burkholderia-caballeronia-paraburkholderia*, *Ralstonia*, and *Staphylococcus* in captive geckos. Many of the species in these genera have been confirmed to be pathogenic in humans and animals; for instance, *Burkholderia pseudomallei* was known to cause melioidoisis (Price et al., 2010); *Ralstonia* was proinflammatory during Parkinson’s disease (Keshavarzian et al., 2015). The higher abundance of opportunistic pathogens in captive geckos was consistent with our prediction 2. In captivity, both dietary shift, constant cohabitation with other congeners, limited range of activity, increased exposure to human-related microbes, and medicine intervention provide pathways for transmission of opportunistic pathogens, leading to differences in the gut microbial communities of captive and wild populations (Jiang et al., 2017; McKenzie et al., 2017). Nonetheless, the causes of differences in the gut microbiota between wild and captive geckos were unknown, since environmental microbes and dietary data were not collected. In addition, our result found total length was significantly positively correlated with these taxa (Supplementary Table 5). Thus, it is difficult to determine whether these bacteria, which are pathogenic to other animals, have an adverse effect on the geckos. More data are needed for further study.

Firmicutes and Bacteroidetes have an impact on the host’s metabolism and immune function mechanisms (Thomas et al., 2011; Zhang et al., 2019). Firmicutes can help to digest and absorb proteins and other nutrients (Kaakoush, 2015; Bernini et al., 2016; Berry, 2016). Most species of Bacteroidetes contribute to the degradation of carbohydrates and proteins (Fernando et al., 2010; Jami et al., 2014). The Firmicutes to Bacteroidetes (F/B) ratio has been studied in both humans and animals and appears to be related to host obesity (Ley et al., 2006; Turnbaugh et al., 2006; Vebo et al., 2016). An increased F/B ratio in gut microbiota indicates a greater energy harvesting capacity for hosts (Turnbaugh et al., 2006; Murphy et al., 2010). In our study, the F/B ratio in the gut microbiota of wild geckos was higher than that of captive geckos. While the diet of captive geckos includes *Z. morio* and *T. molitor*, with high protein and fat content, the diet of wild geckos remains unclear due to limiting conditions in the field. The higher F/B ratio in the wild geckos indicates gut microbiota more efficient at digesting food to help hosts obtain energy in wild populations—a favorable adaptive strategy for wild geckos that survive in harsh natural environments (Jami et al., 2014; Wu et al., 2021). Furthermore, the KEGG pathway analysis revealed that metabolism, genetic information processing, and organismal systems pathways were significantly enriched in wild geckos, providing further evidence that wild geckos may improve adaptability through the strong metabolic potential provided by their gut microbiota.

In summary, there were significant differences between captive and wild geckos in their respective gut microbiota community structures and diversity. Captive geckos had a higher diversity of gut microbiota, but more pathogenic bacteria, while wild geckos had a higher F/B ratio and more metabolism, genetic information processing, and organismal systems pathways. These differences are probably related to differences in living environments and diets of the two gecko populations. Our results could inform researchers in their efforts to further understand the relationship between the gut microbiota of geckos and their living environments and contribute to the comprehensive protection and management of this species in the future.

## Data Availability Statement

The datasets presented in this study can be found in online repositories. The names of the repository/repositories and accession number(s) can be found below: NCBI BioProject - PRJNA817415 (https://www.ncbi.nlm.nih.gov/bioproject/PRJNA817415).

## Ethics Statement

The animal study was reviewed and approved by Laboratory Animal Care and Animal Ethics Committee of Guangxi Normal University.

## Author Contributions

ZC and ZW contributed to conception and design of the study. SY and YTL organized the database. CH performed the statistical analysis. ST and YHL wrote the first draft of the manuscript. All authors contributed to manuscript revision, read, and approved the submitted version.

## Conflict of Interest

The authors declare that the research was conducted in the absence of any commercial or financial relationships that could be construed as a potential conflict of interest.

## Publisher’s Note

All claims expressed in this article are solely those of the authors and do not necessarily represent those of their affiliated organizations, or those of the publisher, the editors and the reviewers. Any product that may be evaluated in this article, or claim that may be made by its manufacturer, is not guaranteed or endorsed by the publisher.

## Footnotes

1
http://www.drive5.com/uparse/

2
http://sourceforge.net/projects/rdp-classifier/

3
https://www.arb-silva.de/

4
https://www.mothur.org/wiki/Download_mothur

5
http://qiime.org/install/index.html

6
http://huttenhower.sph.harvard.edu/galaxy/root?tool_id=lefse_upload

7
https://github.com/picrust/picrust2/
