# Supplementary figures and images for: Comparison of Gut Microbiota Diversity Between Captive and Wild Tokay Gecko (Gekko gecko)
Source: Front Microbiol. 2022 Jun 17;13:897923. doi: 10.3389/fmicb.2022.897923 (PMC9248866; doi:10.3389/fmicb.2022.897923)

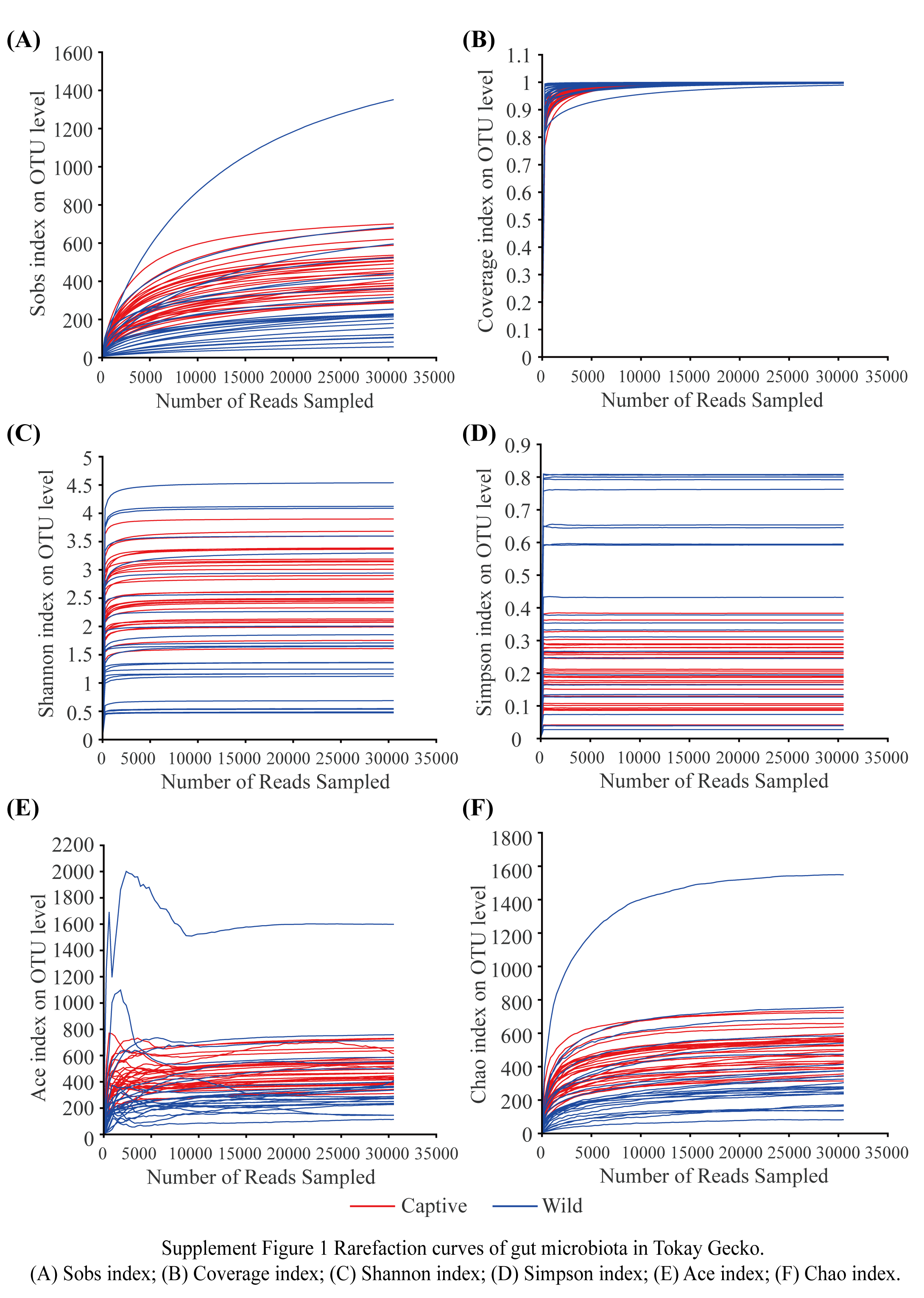

Supplement: Supplementary file 1 [file Image_1.TIF]

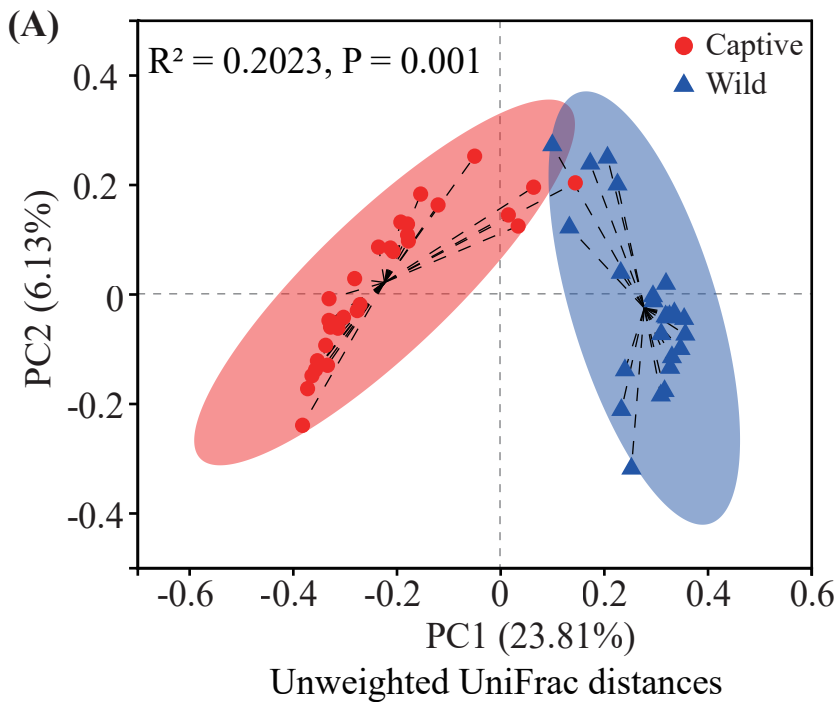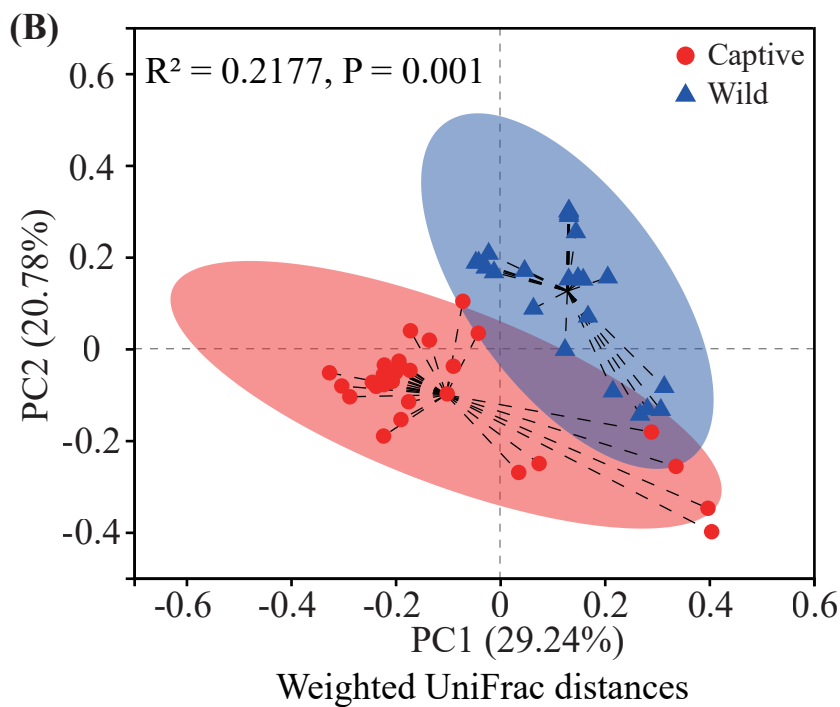

Supplement: Supplementary file 2 [file Image_3.pdf]

(A)

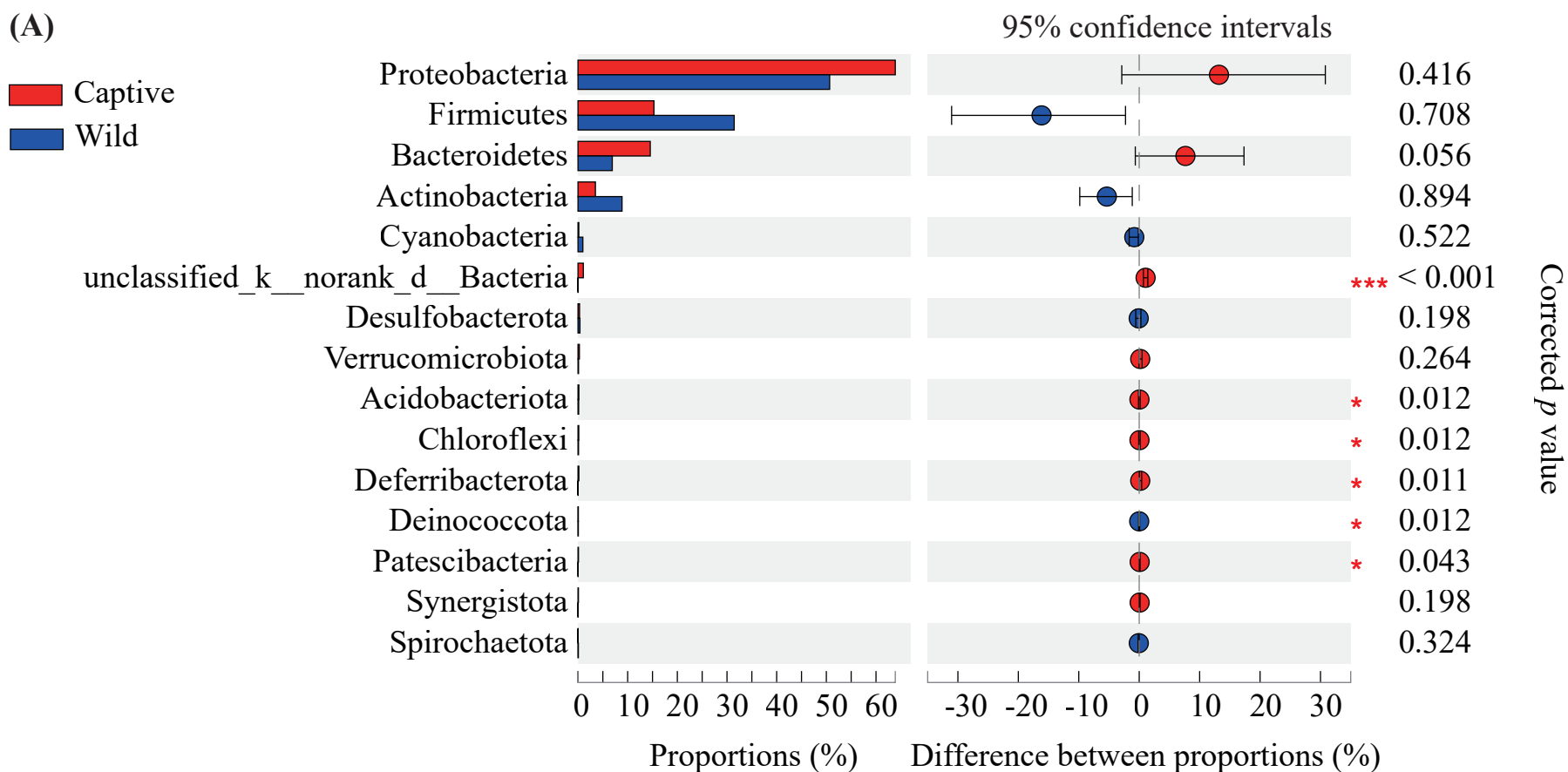

(B)

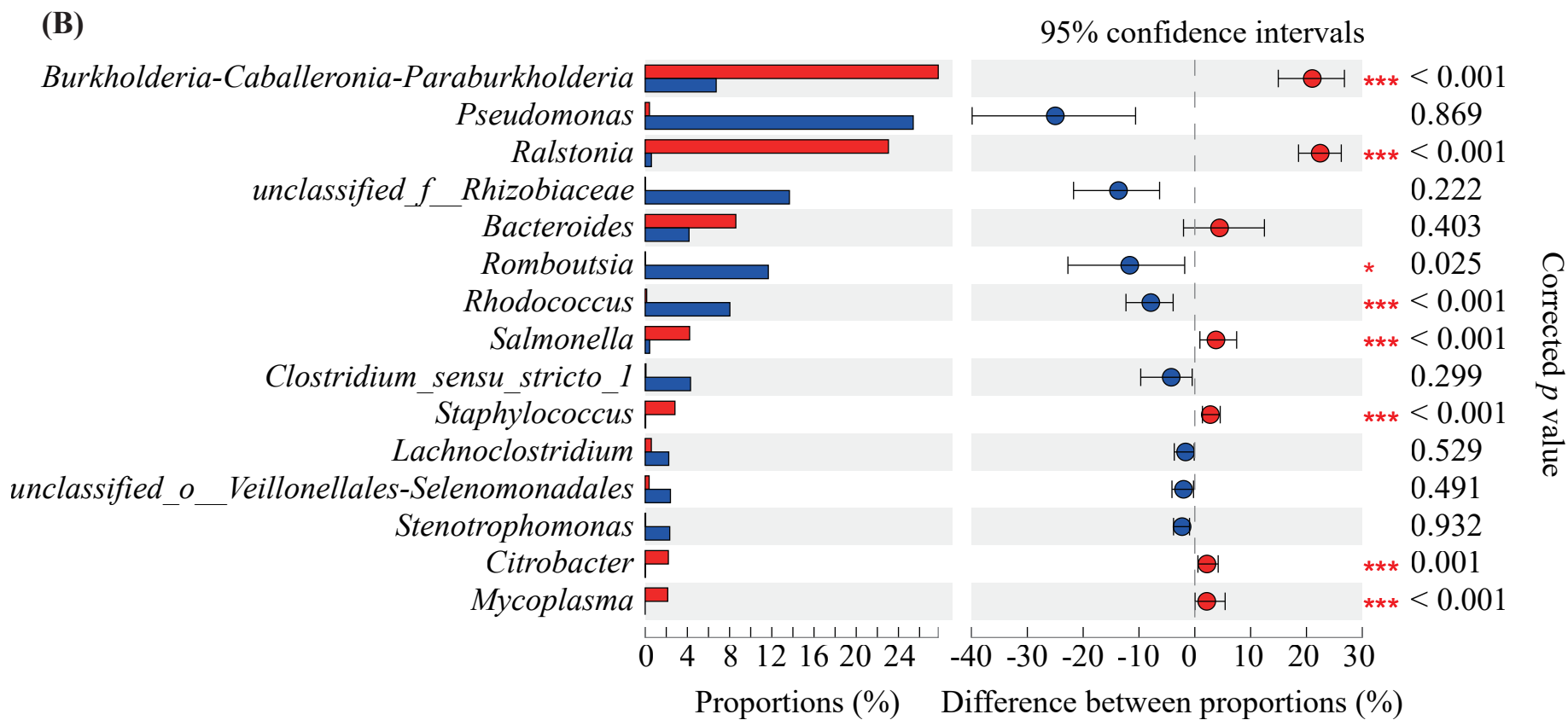

Supplement: Supplementary file 3 [file Image_5.pdf]

(A)

Heatmap of Pathway Level 1

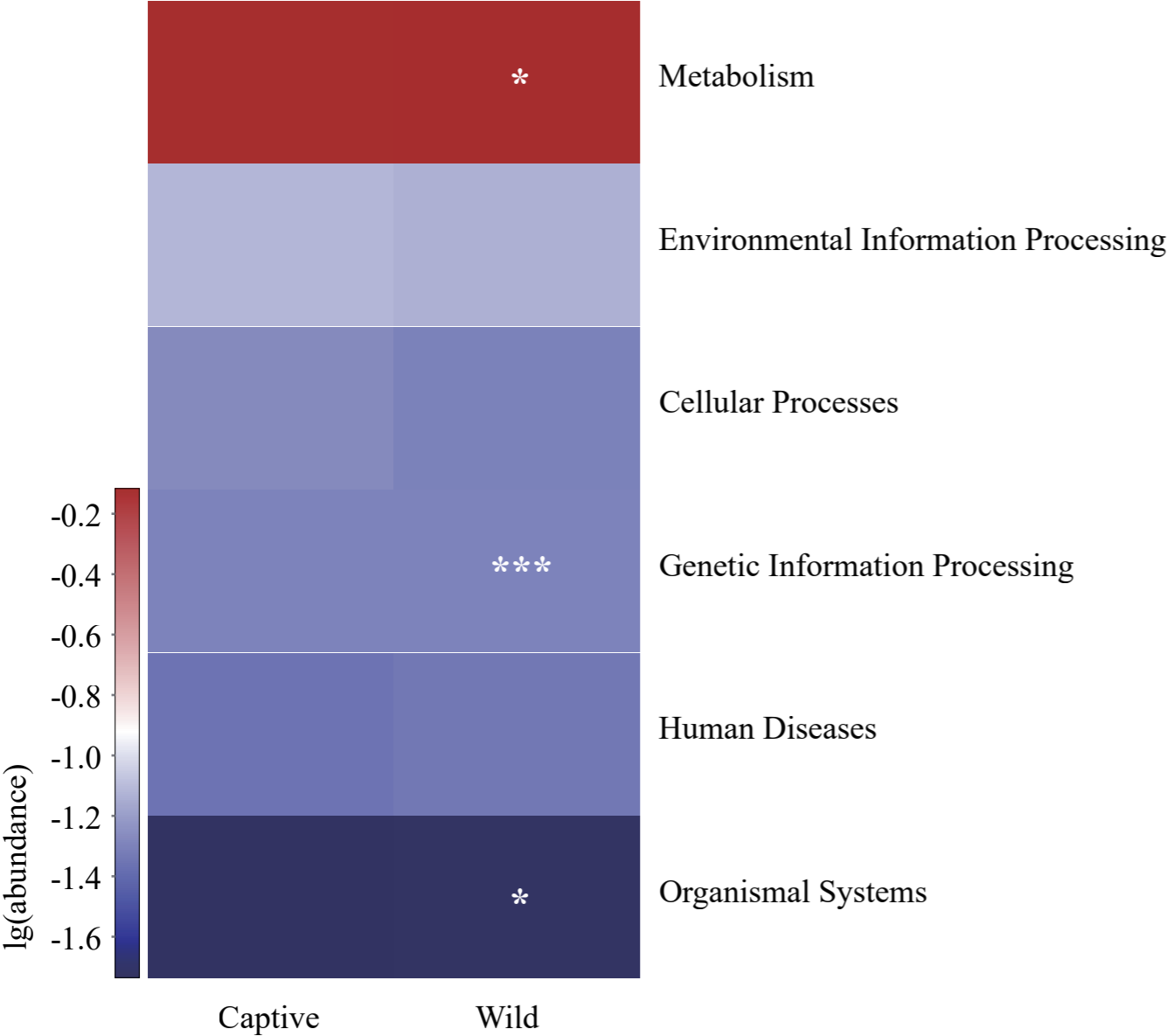

(B)

Heatmap of Pathway Level 2

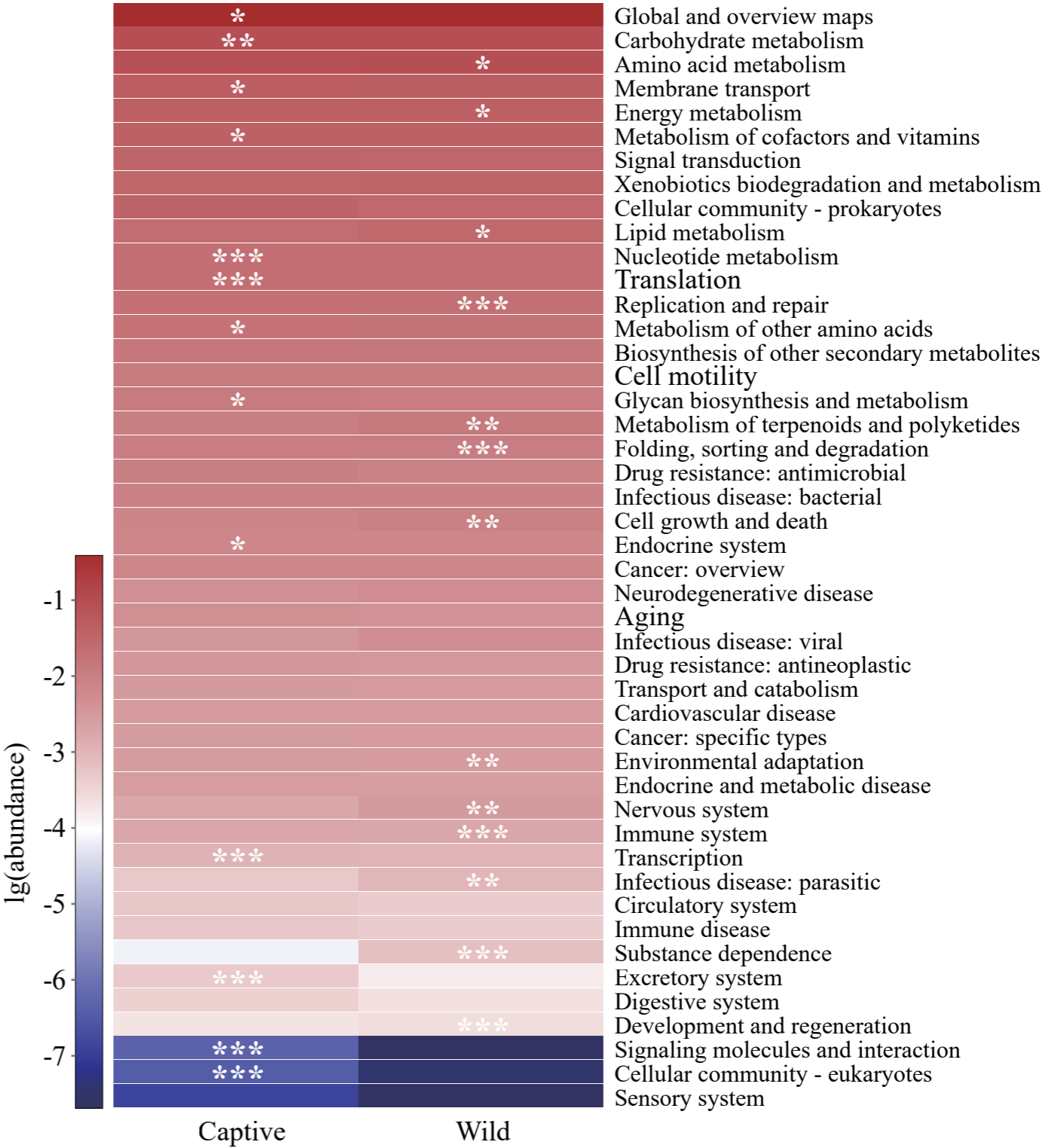

Supplement: Supplementary file 5 [file Image_7.pdf]
