# Supplementary material for: Comparison of Gut Microbiota Diversity Between Captive and Wild Tokay Gecko (Gekko gecko)
Source: Front Microbiol. 2022 Jun 17;13:897923. doi: 10.3389/fmicb.2022.897923 (PMC9248866; doi:10.3389/fmicb.2022.897923)
Supplement: Supplementary file 4 [file Image_6.pdf]

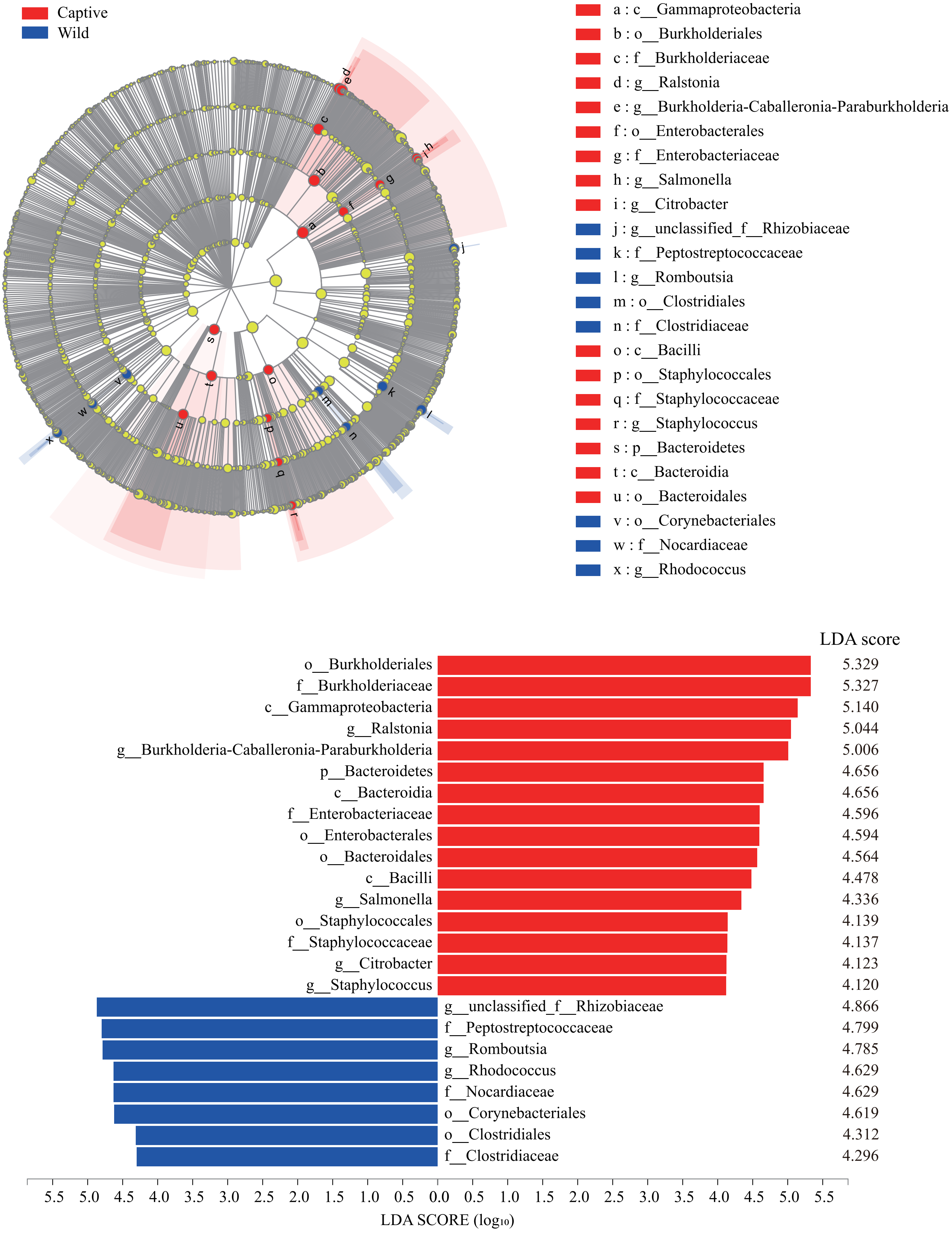
Captive
Wild
a : c__Gammaproteobacteria
b : o__Burkholderiales
c : f__Burkholderiaceae
d : g__Ralstonia
e : g__Burkholderia-Caballeronia-Paraburkholderia
f : o__Enterobacterales
g : f__Enterobacteriaceae
h : g__Salmonella
i : g__Citrobacter
j : g__unclassified_f__Rhizobiaceae
k : f__Peptostreptococcaceae
l : g__Romboutsia
m : o__Clostridiales
n : f__Clostridiaceae
o : c__Bacilli
p : o__Staphylococcales
q : f__Staphylococcaceae
r : g__Staphylococcus
s : p__Bacteroidetes
t : c__Bacteroidia
u : o__Bacteroidales
v : o__Corynebacteriales
w : f__Nocardiaceae
x : g__Rhodococcus
LDA score
o__Burkholderiales 5.329
f__Burkholderiaceae 5.327
c__Gammaproteobacteria 5.140
g__Ralstonia 5.044
g__Burkholderia-Caballeronia-Paraburkholderia 5.006
p__Bacteroidetes 4.656
c__Bacteroidia 4.656
f__Enterobacteriaceae 4.596
o__Enterobacterales 4.594
o__Bacteroidales 4.564
c__Bacilli 4.478
g__Salmonella 4.336
o__Staphylococcales 4.139
f__Staphylococcaceae 4.137
g__Citrobacter 4.123
g__Staphylococcus 4.120
g__unclassified_f__Rhizobiaceae 4.866
f__Peptostreptococcaceae 4.799
g__Romboutsia 4.785
g__Rhodococcus 4.629
f__Nocardiaceae 4.629
o__Corynebacteriales 4.619
o__Clostridiales 4.312
f__Clostridiaceae 4.296
5.5 5.0 4.5 4.0 3.5 3.0 2.5 2.0 1.5 1.0 0.5 0.0 0.5 1.0 1.5 2.0 2.5 3.0 3.5 4.0 4.5 5.0 5.5
LDA SCORE (log10)
