# Supplementary material for: Comparison of Gut Microbiota Diversity Between Captive and Wild Tokay Gecko (Gekko gecko)
Source: Front Microbiol. 2022 Jun 17;13:897923. doi: 10.3389/fmicb.2022.897923 (PMC9248866; doi:10.3389/fmicb.2022.897923)
Supplement: Supplementary file 7 [file Table_2.docx]

**Supplement Table 2 Sequencing information statistics of Tokay gecko (*Gekko gecko*) gut microbiota**

| Groups | Samples | Raw Sequences | Effective Sequences | Average Length |
| --- | --- | --- | --- | --- |
| All samples | 54 | 2603017 | 2504574 | 420.66 |
| Mean |  | 48204.02 | 46381.00 | 420.66 |
| Standard Deviation |  | 9796.15 | 8897.09 | 7.13 |
| Captive samples | 30 | 1384266 | 1340078 | 424.33 |
| Mean |  | 46142.20 | 44669.27 | 424.33 |
| Standard Deviation |  | 11672.87 | 11012.17 | 1.90 |
| Wild samples | 24 | 1218751 | 1164496 | 416.07 |
| Mean |  | 50781.29 | 48520.67 | 416.07 |
| Standard Deviation |  | 6071.12 | 4572.31 | 8.56 |
| Captive | N1 | 41711 | 40009 | 425.17 |
|  | N2 | 57705 | 56854 | 423.69 |
|  | N3 | 46391 | 45346 | 426.20 |
|  | N4 | 36135 | 35181 | 423.68 |
|  | N5 | 31611 | 30572 | 423.98 |
|  | N6 | 66788 | 62575 | 422.67 |
|  | N7 | 48748 | 47334 | 420.85 |
|  | N8 | 55164 | 50966 | 423.57 |
|  | N9 | 44593 | 43844 | 426.48 |
|  | N10 | 36576 | 35673 | 425.72 |
|  | N11 | 46178 | 44498 | 425.19 |
|  | N12 | 38981 | 37144 | 425.30 |
|  | N13 | 48502 | 47586 | 426.41 |
|  | N14 | 40989 | 40268 | 427.53 |

Supplement Table 2 (Continued)

| Groups | Samples | Raw Sequences | Effective Sequences | Average Length |
| --- | --- | --- | --- | --- |
| Captive | N15 | 40822 | 40261 | 423.88 |
|  | N16 | 33175 | 32071 | 424.40 |
|  | N17 | 43819 | 42160 | 421.29 |
|  | N18 | 56144 | 54875 | 424.46 |
|  | N19 | 50837 | 49923 | 427.12 |
|  | N20 | 49261 | 48581 | 427.05 |
|  | N21 | 50890 | 49548 | 425.66 |
|  | N22 | 31650 | 30698 | 423.34 |
|  | N23 | 32560 | 31399 | 421.98 |
|  | N24 | 34740 | 33904 | 424.35 |
|  | N25 | 60907 | 58346 | 421.62 |
|  | N26 | 51258 | 50240 | 426.36 |
|  | N27 | 73970 | 70922 | 423.13 |
|  | N28 | 69053 | 65673 | 420.54 |
|  | N29 | 31680 | 30726 | 423.49 |
|  | N30 | 33428 | 32901 | 424.73 |
| Wild | W1 | 52184 | 49279 | 413.11 |
|  | W2 | 43880 | 43610 | 404.98 |
|  | W3 | 51609 | 50415 | 428.83 |
|  | W4 | 42828 | 42270 | 405.92 |
|  | W5 | 50892 | 50229 | 428.68 |
|  | W6 | 42499 | 42067 | 428.69 |
|  | W7 | 50744 | 50242 | 412.72 |
|  | W8 | 49778 | 49199 | 428.39 |
|  | W9 | 49395 | 46555 | 412.98 |
|  | W10 | 59187 | 52068 | 421.11 |
|  | W11 | 47165 | 46641 | 409.04 |
|  | W12 | 45860 | 45605 | 410.61 |

Supplement Table 2 (Continued)

| Groups | Samples | Raw Sequences | Effective Sequences | Average Length |
| --- | --- | --- | --- | --- |
| Wild | W13 | 53533 | 52749 | 411.77 |
|  | W14 | 60138 | 58450 | 428.84 |
|  | W15 | 44196 | 43389 | 405.77 |
|  | W16 | 54624 | 53115 | 426.42 |
|  | W17 | 59761 | 43719 | 411.64 |
|  | W18 | 45405 | 45054 | 411.75 |
|  | W19 | 63919 | 53762 | 413.40 |
|  | W20 | 58422 | 56831 | 425.78 |
|  | W21 | 46991 | 46492 | 404.64 |
|  | W22 | 48587 | 47095 | 415.14 |
|  | W23 | 44574 | 43683 | 413.77 |
|  | W24 | 52580 | 51977 | 411.70 |
